# Leptospirosis: Morbidity, mortality, and spatial distribution of hospitalized cases in Ecuador. A nationwide study 2000-2020

**DOI:** 10.1371/journal.pntd.0010430

**Published:** 2022-05-12

**Authors:** Manuel Calvopiña, Eduardo Vásconez, Marco Coral-Almeida, Daniel Romero-Alvarez, Miguel Angel Garcia-Bereguiain, Alberto Orlando

**Affiliations:** 1 One Health Research Group, Facultad de Medicina, Universidad De Las Américas (UDLA), Quito, Ecuador; 2 Grupo de Bioquimioinformática GBQ, Facultad de ciencias de la salud, Universidad De Las Américas (UDLA), Quito, Ecuador; 3 Biodiversity Institute and Department of Ecology & Evolutionary Biology, University of Kansas, Lawrence, Kansas, United States of America; 4 Instituto Nacional de Investigación en Salud Pública (INSPI), Quito, Ecuador; Medical College of Wisconsin, UNITED STATES

## Abstract

**Background:**

In Ecuador, leptospirosis surveillance involves a mandatory notification of all cases and a hospitalization for severe illness. Morbidity and mortality are, nevertheless, underestimated and contribute directly to the status of leptospirosis as a neglected disease. *Leptospira* spp. is zoonotic in Ecuador with established endemic transmission in the Tropics. Here, we review retrospective national data within the country to aid in control strategies.

**Methodology/Principal findings:**

In a population-based nationwide study, we analysed morbidity, mortality, and spatial distribution on confirmed hospital-discharged leptospirosis cases from 2000–2020 from a publicly accesible National Database, including males and females of all ages. We computed data for the 24 provinces across the four-geoclimatic regions of Ecuador based on seasonal and monthly variations and calculated rates according to age and sex. The spatial distribution was estimated at the level of ecoregions, provinces, and cantons. A total of 2,584 hospitalizations were recorded over all three continental regions in 22 provinces, except Carchi province and the Galapagos Islands. The annual incidence varied from 0.27 to 2.45 cases per 100,000 inhabitants with ages ranging from 1 to 98 years-old and an overall male/female ratio of 1.92:1. Most hospitalizations and deaths occurred in males ages 25–34 years. We registered 79 fatalities (3.06%); the highest mortality rate was 0.05 per 100,000 inhabitants. More cases clustered in the tropical cantons of central and north of the Coast and in the southern Amazon when compared to the Andes.

**Conclusions and significance:**

Our findings evidence leptospirosis endemicity and pinpoint the highest incidence within resource-poor tropical settings. The highest incidence occurred in males of adult age, with those also exhibiting the highest mortality. The national incidence rate was stable, but peaks occurred intermittently during the rainy seasons. Thus, strategies aimed at leptospirosis monitoring and control involving the application of preventive measures should consider this season and the aforementioned high-risk groups.

## Author summary

Leptospirosis is a neglected tropical disease poorly characterized in Latin American countries like Ecuador. Although this zoonotic disease is considered by the Epidemiology Department of the Ministry of Public Health to be of mandatory notification, an updated document on the patterns of morbidity and mortality or a control strategy do not yet exist. In the present study, we carried out a retrospective analysis of Ecuadorian national data (INEC) on hospitalized cases and deaths from 2000 through 2020 to estimate the trends of morbidity and mortality, and the spatial distribution of this disease throughout its four geoclimatic regions (Pacific coastal, Andes, Amazon, and the Galapagos Islands). The INEC recorded only hospitalized cases, meaning those with severe illness. The spatial distribution was estimated at the ecoregion, province, and canton levels. With a total number of 2,584 hospitalizations, an annual incidence ranging from 0.27 to 2.45 cases per 100,000 inhabitants, and a fatality rate of 3.06% during the period of study. Our findings evidence the endemicity of leptospirosis, particularly within the neglected tropical settings in the Ecuadorian Coast and Amazon regions. Despite the wide geographic distribution of leptospirosis and its severity in terms of incidence, morbidity, and mortality, leptospirosis is not yet considered a “tool-ready” disease for control/elimination and research initiatives.

## Introduction

Leptospirosis is a neglected tropical disease caused by spirochetes of the genus *Leptospira* with a worldwide distribution. The consideration of leptospirosis as an emerging public-health concern is due to its morbidity and mortality [1,2]. Infections are zoonotic and are acquired through contact with infected urine from domestic or wild animals, or through a contaminated environmental source. Leptospirosis is mainly seen in vulnerable populations living in tropical climates within developing countries [3,4].

The infection does occur in temperate climates where the incidence has been estimated to vary between 0.1 and 1.0 per 100,000 population, but the incidence may be 10-fold greater in tropical settings [2]. Leptospirosis causes an estimated 1.03 million human infections and 58,900 deaths annually [2]. In Latin America, the Pan American Health Organization (PAHO) has estimated the number of annual cases at 10,702 of which 40.2% occur in Brazil, 23.6% in Peru, 8.8% in Colombia, and 7.2% in Ecuador [5]. The incidence of human leptospirosis is expected to increase over the coming decades as a consequence of the increase of periurban populations living in poor housing conditions with limited access to basic services along with an increase in the frequency of extreme weather events, consequent to anthropogenic climate change [6].

The severity of the disease depends on the *Leptospira* spp. The *Leptospira* strains infecting humans and animals can be categorized as pathogenic, intermediate, or saprophytic [1]. The saprophytic or biflexa group are nonpathogenic, while the intermediate group may cause a mild but chronic infection. The pathogenic group induces a variety of disease symptoms from mild undifferentiated fever to a severe illness with acute renal failure and pulmonary haemorrhage requiring hospitalization. Fatality rates among confirmed cases range from 5% to 15% [1]. Knowledge of the incidence of infections, including asymptomatic or mild cases, is critical in the epidemiology of the disease in a given country. However, a full description of case numbers and the spatial distribution of hospitalized cases, remains a challenge and has implications in decision-making for leptospirosis management [7].

Ecuador is located in northwest South America at the Equator, with three continental geoclimatic regions (Pacific coastal, Andes, and Amazon) along with a fourth, the Galapagos Islands. Leptospirosis in the country, is considered endemic for animal and human infections with outbreaks reported in urban, suburban, and rural populations, particularly during rainy seasons [8–11]. The Ecuadorian Ministry of Health (MoH) estimated an annual incidence of 1 case per 100,000 population at the national level with an average of 547 new cases reported between 2016 and 2020, primarily from cantons and provinces of the Pacific coastal region [12]. Data from the MoH-Integrated Epidemiological Surveillance System (SIVE-ALERTA) has been used to describe an increase in annually reported cases of leptospirosis from 155 in 2003 to 1,279 in 2012 [13]. SIVE-ALERTA collects cases through surveillance of outpatients with suspected leptospirosis attending a health unit with a later confirmation using laboratory testing or by epidemiological linkage [14]. In a recent publication, an analysis using the same above-cited database indicated that the incidence rates decreased from 3.3 to 0.8 cases per 100,000 population between 2013 and 2018 [15]. This database did not report hospitalized cases or mortality.

The World Health Organization (WHO) and various governments have called for data on the epidemiology of neglected tropical diseases in order to develop or improve prevention and control strategies based on updated evidence to identify priorities in decision-making [3,14]. Country-level estimates, however, can have variations within each of the national estimates, needing assessments at subnational levels, especially in countries with different geoclimatic regions and islands. Because of the lack of information on leptospirosis in Ecuador at the subnational level, we have analysed data for the incidence and mortality of the human cases registered by the INEC over a 21-year period (2000–2020) and estimated the spatial distribution even at canton level of hospitalized *Leptospira* spp. infections in order to inform local decision-making and policy.

## Materials and methods

### Country of study

**Fig 1:**
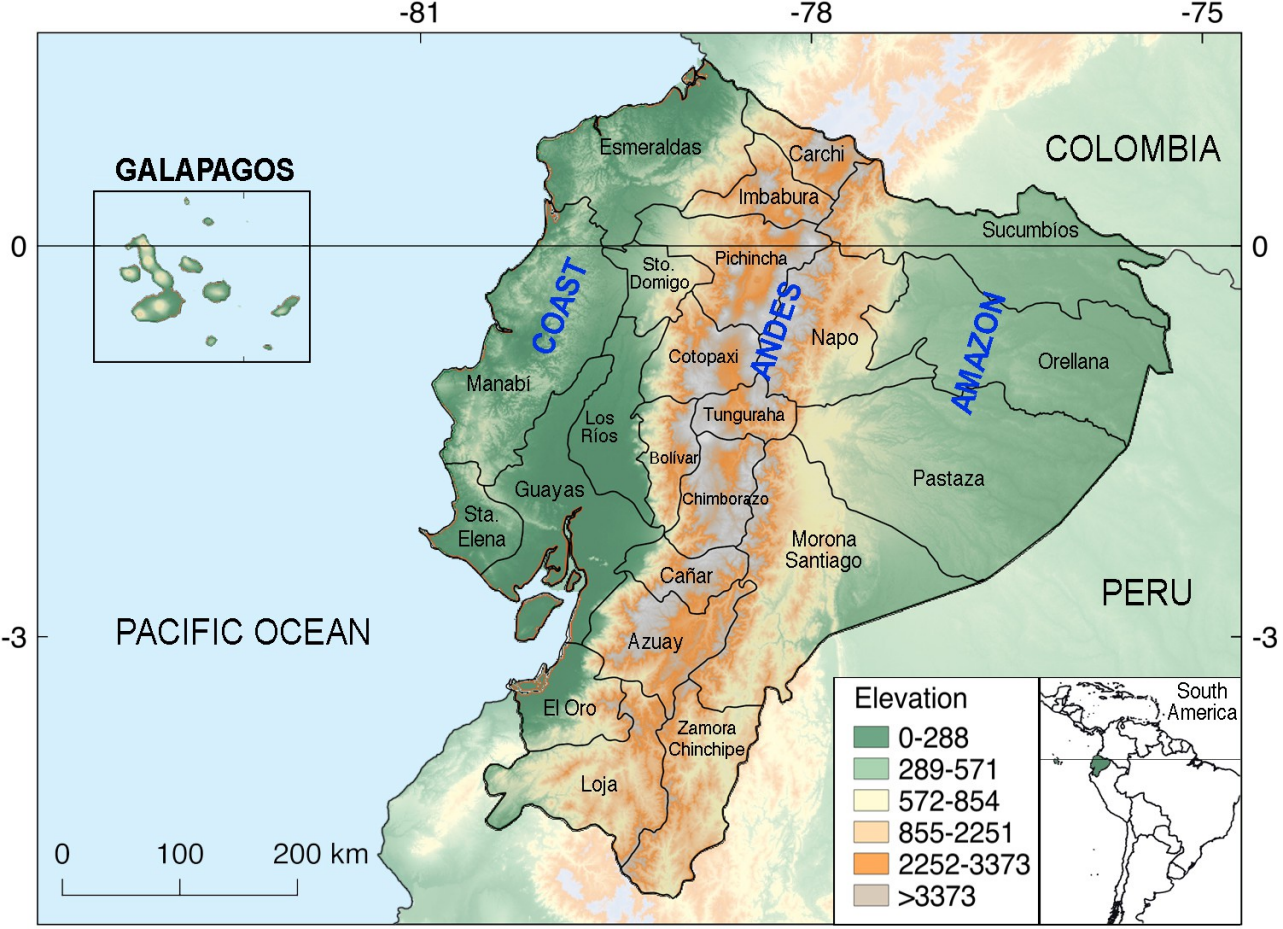
Geographic map of Ecuador. Administratively, Ecuador is divided in 24 provinces containing 224 cantons with differing geographic, socioeconomic, and cultural characteristics. Seven provinces lie on the Coast, ten in the Andes, and six in the Amazon. The Galapagos Islands are in the Pacific Ocean at 1,369 km from the Ecuadorian coast and have dry and warm weather. Cantons are second-order administrative divisions, within the provinces. The map was created with QGIS 2.18. (Geographic Information System, Open Source Geospatial. Foundation Project. http://qgis.osgeo.org). The box to the upper left encloses the Galapagos Islands, while the box to the lower right provides a color-coded key to the land-elevation ranges within the map.

Ecuador, located in northwest South America and covering an area of 283,560 km^2^, is crossed by the Equator and bisected North-South by the Andes mountains, dividing the continental land into three geoclimatic regions in addition to the Galapagos Islands: 1) the Pacific coastal tropical and subtropical lowlands with an average temperature of 25°C and annual rainfall of 1,000 mm, 2) the temperate highland Andes (including the inter-Andean valleys with a subtropical climate) with an average annual temperature ranging from 8 to 22°C, and 3) the tropical Amazon basin, with an average temperature of 28°C and annual rainfall in excess of 5,000 mm (**Fig 1**). Ecuador’s climate involves rainy and dry seasons. In the Coast and Andes regions, the rainy months stretch from October–December through April–May; conversely, rains are constant in the Amazon region though more intense between April and November [16]. Ecuador’s population in 2020 was estimated at 17,510,643 inhabitants; of which total, 8,631,859 live on the Coast, 7,847,136 on the Andes, 956,699 on the Amazon, and 33,042 on the Galapagos Islands; approximately 36% of the population lives in rural areas [17]. The Coast and Amazon lowlands are at a particular risk of flooding during periods of intense rains that may be exacerbated by the El-Niño–Southern-Oscillation phenomenon [18] and because of the growing problems of urbanization and environmental degradation [19].

### Study design and population

We conducted the present cross-sectional and retrospective study to determine the patterns of leptospirosis morbidity, mortality, and spatial distribution using the hospitalized cases for the period 2000 to 2020 (21 years) registered by INEC. The scope of the present study included all the individuals with a confirmed diagnosis of leptospirosis who had been hospitalized throughout the entire continental and island regions of the country.

### Leptospirosis cases and operational definitions by the MoH-Ecuador [14]

#### Suspected cases

All persons with fever accompanied with at least two of the following symptoms: headache, chills, muscle ache, bilateral conjunctival congestion, confusion, with or without jaundice; hemorrhagic manifestations on the skin, nausea, vomiting, or abdominal pain in addition to any epidemiological link in the form of exposure to potentially contaminated water sources and people with occupational-risk activities.

#### Confirmed cases by laboratory analysis

Any patient who met the criteria for a “suspected case” plus a positive microagglutination test (MAT). The indirect test involving the enzyme-linked immunosorbent assay ELISA (detecting IgM-type antibodies) is the most widely used method to screen for acute leptospirosis, two serum samples within an interval of several days are recommended, though any indirect ELISA-positive result must be confirmed by the MAT.

#### Confirmed cases by epidemiological link

“Suspected cases” without a laboratory confirmation but associated with a laboratory-confirmed index case—and therefore potentially exposed to the same source of infection is considered as “confirmed case”.

#### Severe cases

Symptoms of acute pulmonary oedema, acute renal failure, gastrointestinal bleeding, myocarditis without pulmonary disorders, coagulation disorders, and jaundice are considered “severe case” and required hospitalization.

### Source of data and procedure

Leptospirosis is an illness requiring by law immediate notification in Ecuador, where the hospitalization of severe cases is compulsory. We used national surveillance data for hospital admissions and/or discharges retrieved from a publicly accessible database from the INEC, available online [https://www.ecuadorencifras.gob.ec/camas-y-egresos-hospitalarios/]. The database was scanned for information on hospitalized cases and deaths through the use of the International Classification of Diseases (ICD-10) code A27 for leptospirosis (S1 Table). The INEC registers only confirmed cases by laboratory test. Data were stratified by age, sex, discharge status (dead or alive), geographic location (geoclimatic region, provinces, and cantons), and monthly-confirmed cases.

### Data quality control

Data were checked year by year from 2000 to 2020 for accuracy, completeness, and consistency before analysis to ensure data quality. Any data that were not properly documented and not completely registered were excluded from the analysis. A Microsoft Excel spreadsheet was prepared for data recording.

### Statistical methods

To estimate the morbidity and mortality rates, population data were obtained from the INEC with respect to the annual population estimates for the period between 2000 and 2020, based on the Ecuadorian national census of 2010 [17]. The incidence rates were estimated per 100,000 inhabitants. The case-fatality rates were estimated as the ratio of the number of deaths reported to the total number of cases reported. The associations of leptospirosis occurrence with variables such as sex and age were assessed by means of Pearson´s Chi-squared analysis. Additionally, we tested if any difference occurred between monthly periods within the three continental ecoregions Coast, Andes, and Amazon. Because the country lies on the Equator, seasons were divided into either dry or rainy, roughly overlapping with semestral cycles; we compared the total number of cases throughout the entire study period during the first six months of the year versus the next six months using a Wilcoxon signed-rank test with a moving window considering one month at a time to make iterative comparisons, to assess all possible semestral combinations (*i*. *e*., six). The data were analysed by means of R software 3.6.3 [https://cran.r-project.org/] and Microsoft Excel. Statistical significance was defined as *p* values <0.05.

### Spatial analysis

First, we calculated the cumulative incidence of hospitalized cases per geoclimatic region and province in a choropleth map. Then, we used a Poisson regression to assess the potential statistical differences in the number of cases within geoclimatic regions and provinces and calculated the relative risk (RR) at the corresponding administrative level. Next, we conducted a spatial-cluster analysis in SATSCAN V9.6 [SaTScan TM. Software for the spatial, temporal, and space-time scan statistics (https://www.satscan.org/] to identify cantons (*i*. *e*., spatial-analytical units) with statistically significant higher incidences. To perform this analysis, we used the number of inpatients, the spatial coordinates (centroids), and the total population of each canton. We compared the hospitalized cases using a Poisson distribution. Space clustering was assessed by a comparison of the incidence-rate ratio of inpatients within a specific geographical area in contrast to the incidence-rate ratio expected for hospitalized cases considering that incidences were randomly distributed across the country. Finally, we used a likelihood-ratio test and Monte-Carlo simulations with 999 runs to test the statistical significance of space clusters identified via *p* values. A cluster was identified as significant if the *p* values were <0.05 [20].

### Ethics statement

The INEC database is anonymized and publicly available. This article is a review of INEC data and does not require a bioethics approval. The shape files used for all the maps in this work were obtained from the INEC portal in a manner following their licensing requirements. All the maps were created and designed through the use of the QGIS version 2.18 ‘Las Palmas’ [QGIS Geographic Information System. QGIS Association. http://www.qgis.org.] and Adobe Photoshop Elements.

## Results

### Trends of incidence and mortality (2000–2020)

**Fig 2:**
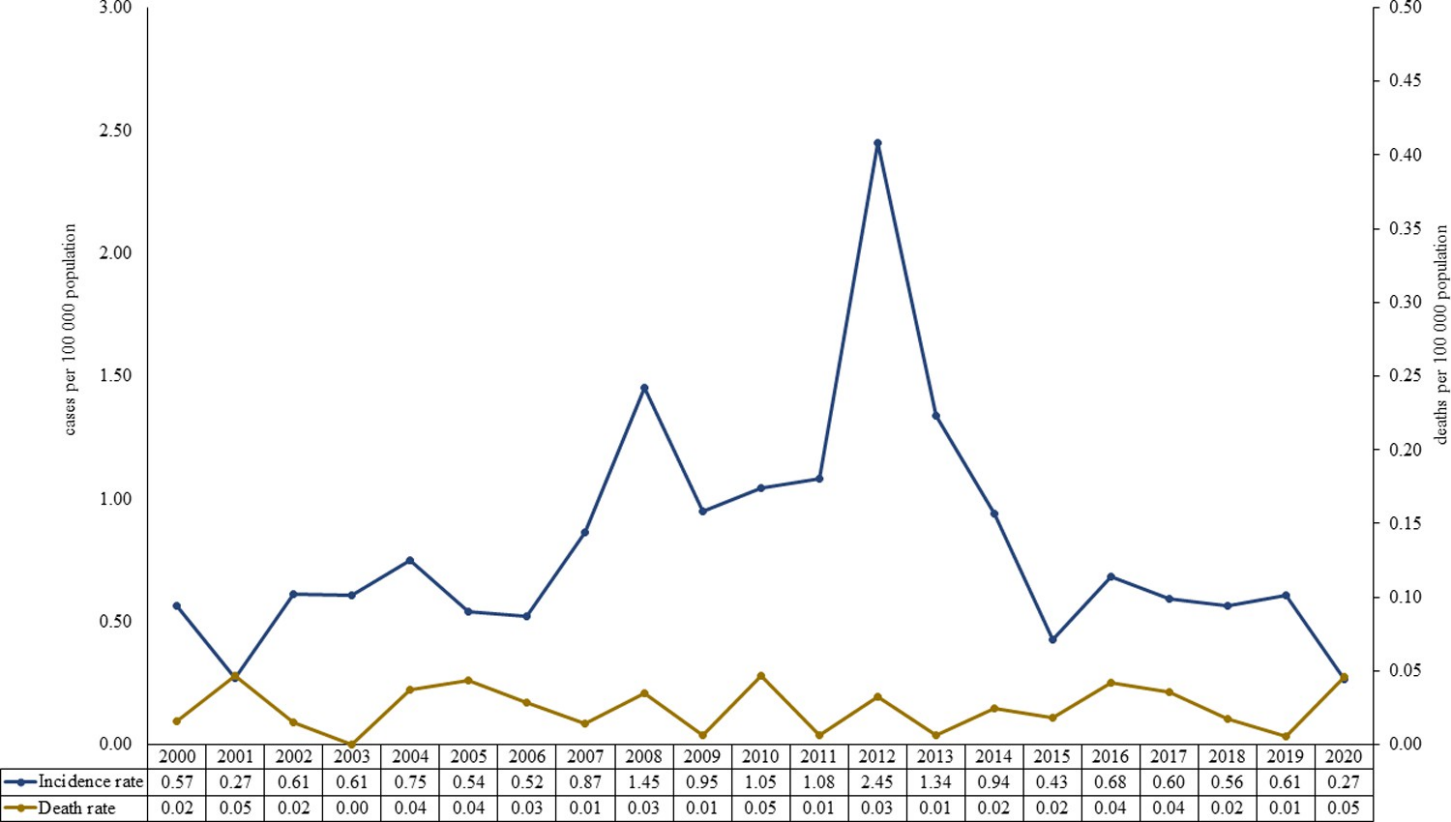
Annual incidence and mortality of leptospirosis (2000–2020). In the figure, the cases per 100,000 population are plotted on the *ordinate* as a function of the years on the *abscissa* for the incidence rate (blue) and the death rate (yellow). The box with the key to the colors at the lower left also marks the respective data for each year in the two rows underneath the *abscissa*.

A total of 2,584 hospitalized cases were registered in the INEC, with a median of 123 cases/year over all three continental geoclimatic regions, in 22 of the 24 provinces except for Carchi and the Galapagos Islands. The ages ranged from 1 to 98 years (mean 30.7, median 27). Annually, the incidence rate varied from 0.27 to 2.45 per 100,000 inhabitants for 2001 and 2012, respectively. In total, 79 deaths associated with leptospirosis were reported, giving a cumulative case-fatality rate of 3.06%. Most of the cases (65.9%) and deaths (64.8%) were reported in males. The age range of fatal cases was 14 to 91 years (**Fig 2**).

### Leptospirosis according to sex and age groups

**Fig 3:**
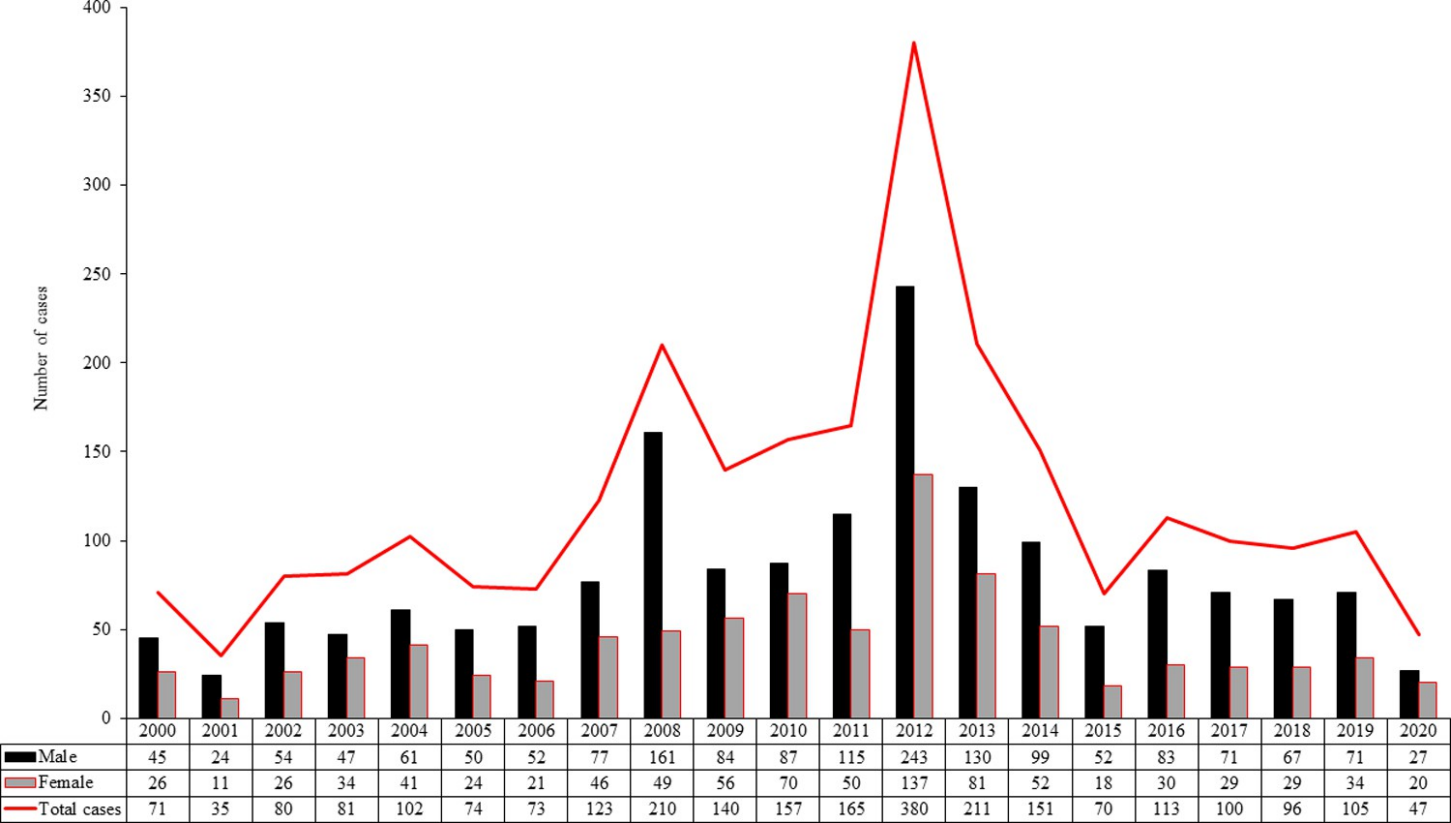
Number of cases of leptospirosis by year and sex (2000–2020). In the figure, the number of cases is plotted on the *ordinate* as a function of the years on the *abscissa* for males (black bars), females (gray bars), and the total (red line). The box with the key to the colors at the lower left also marks the respective data for each year in the two rows underneath the *abscissa*.

**Fig 3** summarizes the number of inpatients by calendar year and sex. Most hospitalized cases corresponded to males (males 65.7% *vs*. females 34.3%, *p* <0.001) giving a male to female ratio of 1.92:1.00.

**Fig 4:**
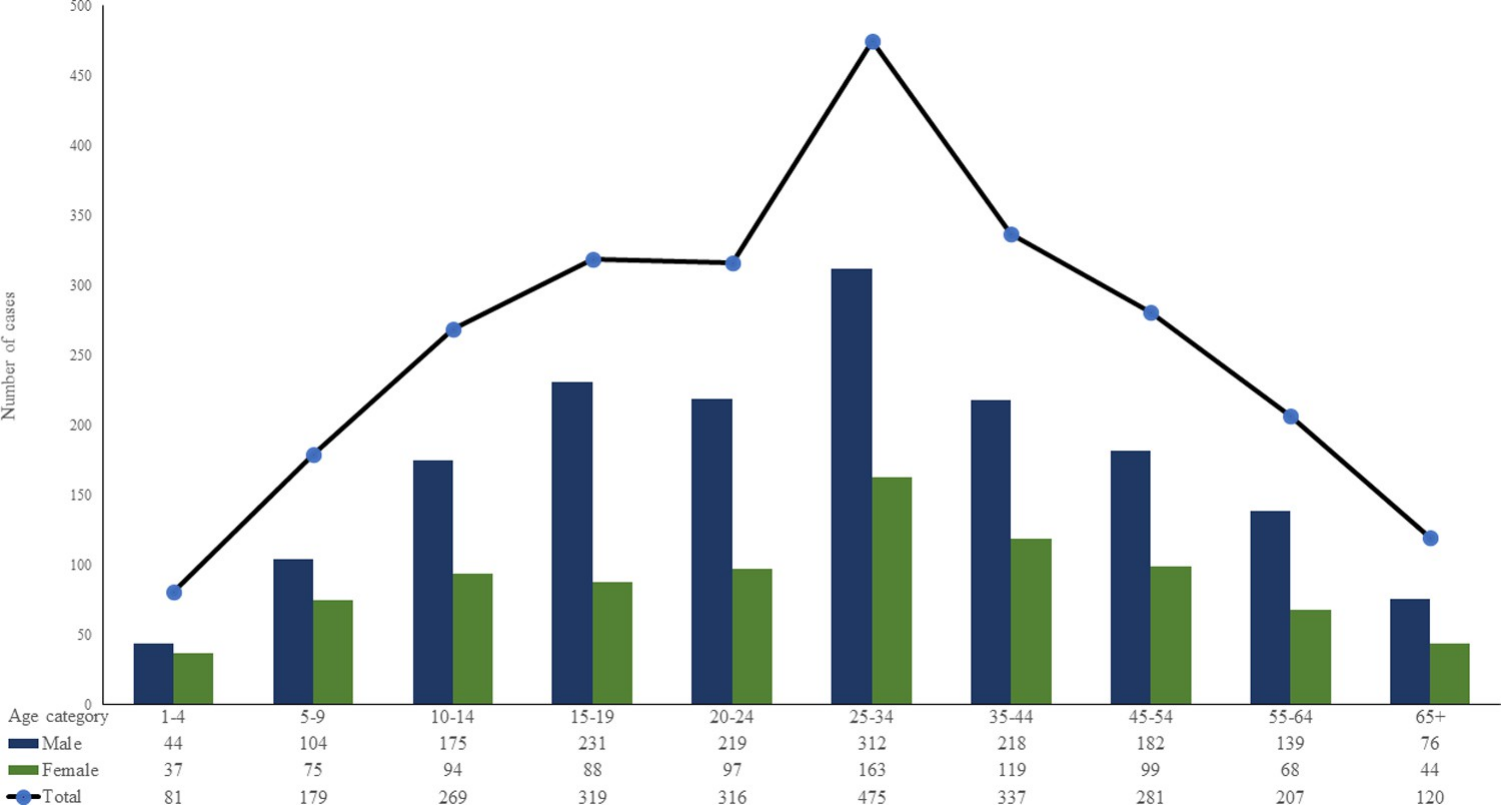
Distributions of the 2,584 cases over the 21-year period divided by age groups and sex. Cases were reported over all ages (range, 1 to 98 years; mean/median, 31/27 years), but were least frequent between birth and 4 years of age. Male and females aged 25 to 34 years, were the most frequently hospitalized. A statistically significant association was found between the frequency of cases and the age category (p <0.001), with the age categories of 15–19 and 25–34 years being the ones with the highest rates of infection compared to age categories 1–4 and over 65 years, having the lower infection rates.

Distributions of the 2,584 cases over the 21-year period divided by age groups and sex are shown in **Fig 4**.

### Geographical distribution (2000–2020)

#### Incidence and relative risk by ecoregions and provinces

**Fig 5:**
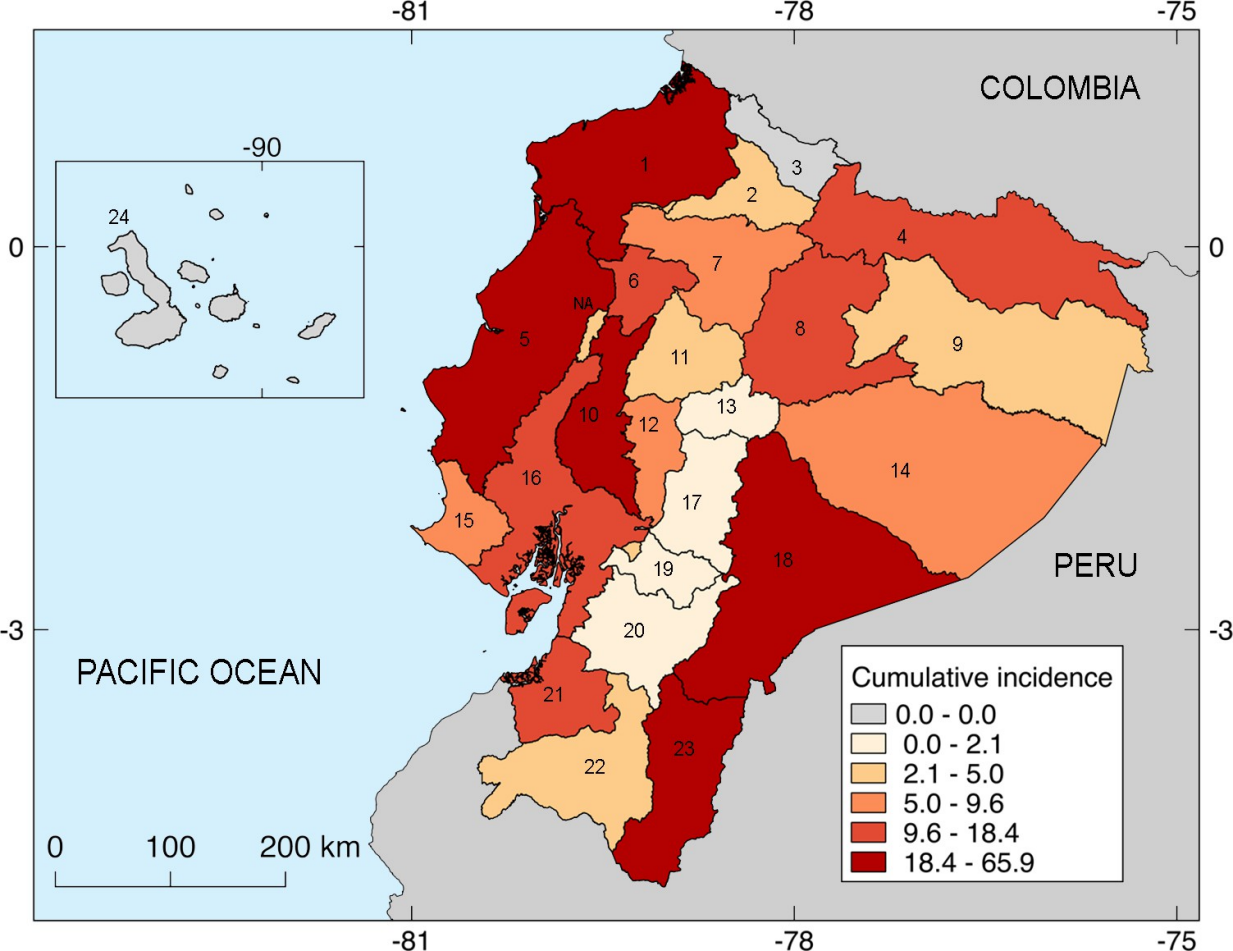
Cumulative incidence of hospitalized cases per 100,000 population by province. The incidence was calculated with the total population of 2020 as denominator. The color ramp on the lower right comprises the spectrum of quintiles for the total incidence. The numbers in the square brackets in the following indicate leptospirosis case numbers corresponding to the numbered provinces on the map: Esmeraldas [No. 1: 186], Imbabura [No. 2: 11], Carchi [No. 3: 0], Sucumbíos [No. 4: 33], Manabí [No. 5: 1029], Santo Domingo de los Tsáchilas [No. 6: 46], Pichincha [No. 7: 171], Napo [No. 8: 23], Orellana [No. 9: 8], Los Ríos [No. 10: 186], Cotopaxi [No. 11: 15], Bolívar [No. 12: 12], Tungurahua [No. 13: 9], Pastaza [No. 14: 9], Santa Elena [No. 15: 30], Guayas [No. 16: 502], Chimborazo [No. 17: 7], Morona Santiago [No. 18: 91], Cañar [No. 19: 3], Azuay [No. 20: 16], El Oro [No. 21: 90], Loja [No. 22: 23], Zamora Chinchipe [No. 23: 79], and the Galapagos [No. 24: 0]. The map was created by QGIS 2.18.

The INEC recorded cases in the three continental geoclimatic regions. Most cases were reported in the Coast 2,070/2,584 (80.1%), followed by the Andes (10.5%) and the Amazon (9.4%). No case was reported from the Galapagos Islands. The Coast had the highest incidence with an average annual rate of 1.12 per 100,000 population. A comparison of the occurrence between the geoclimatic regions indicated a significant positive association between the Coast and the Amazon regions (RR = 5.7 and 5.4, respectively; *p* <0.001) as opposed to the Andes. Fig 5 delineates the cumulative incidence rates by province: the provinces of Esmeraldas (1), Manabí (5), and Los Rios (10) within the Coast and the provinces of Morona Santiago (18) and Zamora Chinchipe (23) within the Amazon had an estimated cumulative incidence of >18.4 per 100,000 population. Twenty-two of the 24 provinces presented at least one case of leptospirosis. We found a significant statistical association (*p* <0.001) of increased cases in 14 of the 22 provinces reported with RRs ranging from 3 to 37 when compared to the rest of the country. The 3 provinces with the highest RRs were: Manabí, Zamora Chinchipe, and Morona Santiago.

#### Spatial analysis by Cantons

**Fig 6:**
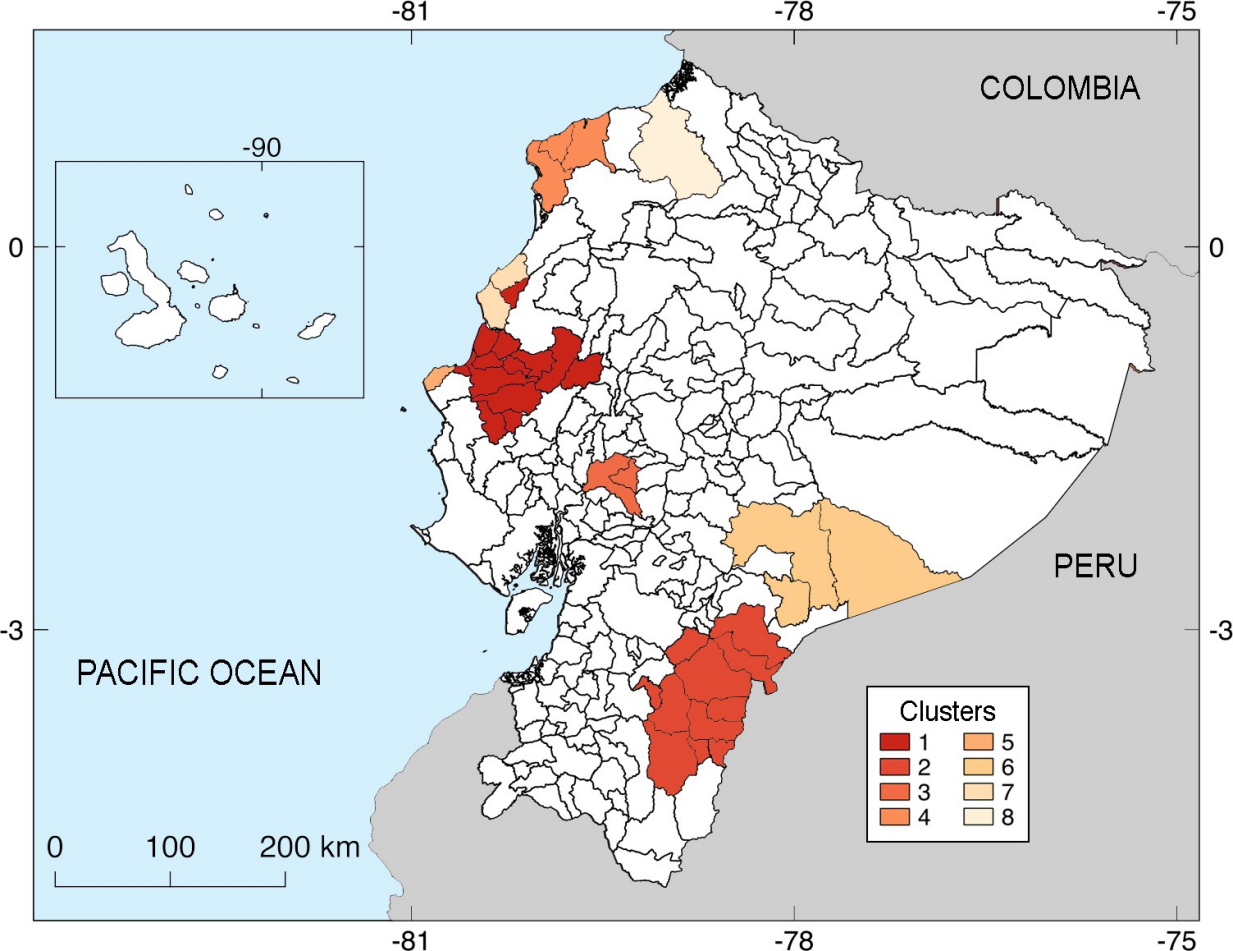
Clusters identified at the canton level of Ecuador based on the cumulative incidence. The color ramp on the lower right comprises the spectrum of spatial clusters for the total incidence. This spatial analysis identified cantons with the highest numbers of hospitalized cases (No. 1, bright red) and those with the lowest number (No. 7, pale orange). Numbers represent the following cantons: **No. 1**: Junín, Bolívar, Tosagua, Portoviejo, Rocafuerte, Santa Ana, Sucre, Pichincha, Jaramijó, Olmedo, 24 de Mayo, and Empalme; **No. 2**: El Pangui, Yantzaza, Gualaquiza, Paquisha, Centinela del Cóndor, Zamora, Oña, Sígsig, San Juan Bosco, and Limón Indanza; **No. 3**: Babahoyo and Montalvo; **No. 4**: Atacames, Esmeraldas, and Muisne; **No. 5:** Manta; **No. 6**: Taisha, Morona, and Logroño; **No. 7**: Jama and San Vicente; **No. 8**: Eloy Alfaro.

For leptospirosis-hospitalized cases, the cluster analysis considering cantons as the administrative unit allowed us to recognize 12 spatial clusters with statistical significance (*p* <0.001). Among these clusters, leptospirosis RRs considering spatial associations of contiguous cases ranged from 10.8 in the principal cluster to 2.78 in the least significant one (Fig 6). From these 12 clusters, Fig 6 indicates eight nonoverlapping cantons, six in the central and north central provinces of the Coast, and two in the southern provinces of the Amazon.

#### Seasonality

**Fig 7:**
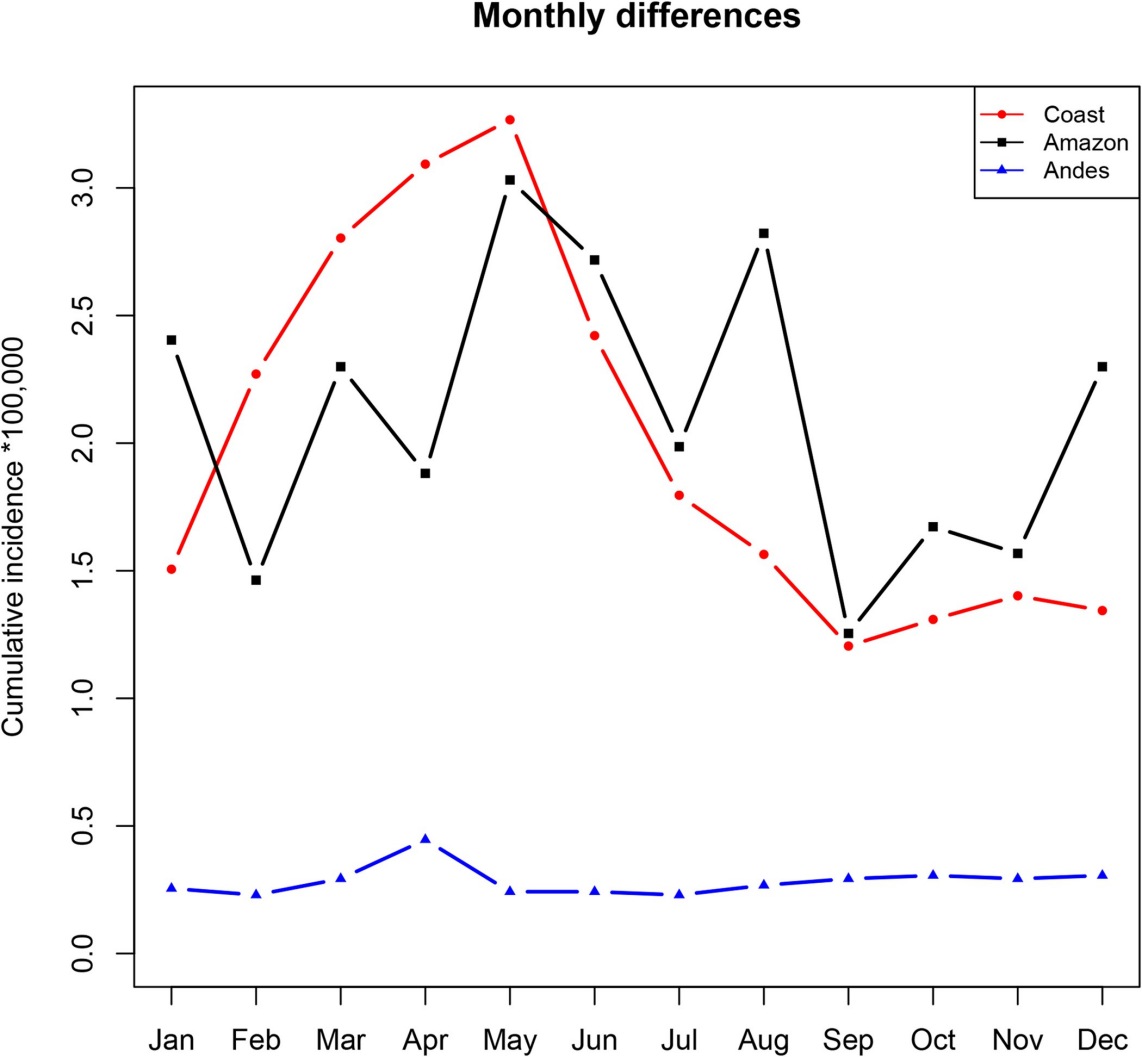
Hospitalized leptospirosis by the month in the three continental geoclimatic regions (2000–2020). In the figure, the cumulative incidence per 100,000 is plotted on the *ordinate* as a function of the months of the year indicated on the *abscissa* for the Coast (red circles), Amazon (black squares), and Andes (blue triangles) ecoregions. An increase in cases occurred for the Coast from January to June. Cases throughout the Amazon increased and decreased during the year. In contrast, the number of hospitalizations in the Andes (blue) was relatively constant over time except for a peak of hospitalizations in April. The incidence was calculated from the total estimated population of Ecuador for 2020 as the denominator per 100,000 inhabitants.

Fig 7 reveals a seasonal pattern of higher numbers of leptospirosis cases for the Coast. By testing differences between six-month intervals between different regions, we found that for the Coast, a pattern of statistical differences obtained upon comparing January–June *vs*. July–December (W = 34, *p* = 0.009), February–July *vs*. August–-January (W = 36, *p* = 0.002), and March–August *vs*. September–February (W = 34, *p* = 0.009). For the Andes and the Amazon, no statistical differences were registered between any of the comparisons.

## Discussion

The present nationwide study reveals that leptospirosis in Ecuador represents a public health concern as evidenced by the disease’s incidence and countrywide distribution. The annual incidence of hospitalizations was high (median of 123 cases/year), and the cumulative case fatality rate was 3.06% in the 21-year study period. Furthermore, hospitalized cases and mortality were registered over the entirety of continental Ecuador, including the temperate region of the Andes. No hospitalized human cases were recorded in the Galapagos Islands, coinciding with a recent report from the Epidemiological-Surveillance-System (SIVE-ALERTA) database [15]; nevertheless, we need to mention that evidence exists of leptospirosis infections in sea lions [21] which may serve as a potential source for the disease to become enzootic among livestock on the islands.

The highest incidence rates were recorded in the Coast and Amazon ecoregions, which are subtropical and tropical lowlands, as other previous studies using SIVE-ALERTA database have reported [13,15], meaning that a positive correlation existed between suspected and/or epidemiology-linked cases recorded in SIVE-ALERTA, and the hospitalized cases recorded in the INEC.

Our data indicate that hospitalized cases significantly increased from 2006 to 2012 with the highest peak occurring in 2012; subsequently, a decrease in case numbers from 2012 to 2020 was also registered, though not reaching statistical significance. These data are in agreement with the epidemiologic pattern reported by the SIVE-ALERTA database indicating an increase in leptospirosis cases up to 2013 [13] followed by a decrease from 2013 to 2018 [15], which concordance likewise suggests consistent epidemiologic dynamics reflected by both datasets. Nevertheless, we found a certain amount of disagreement between local reports and the official national data. During 2010–2012, >2,000 serologically confirmed cases of febrile leptospirosis cases were reported by local health authorities in Portoviejo-Manabí province [10], but only 1,784 cases were recorded by SIVE-ALERTA [13]. Hence, a review of the existing outpatient passive-surveillance system is warranted. The main advantage of the INEC database is that only inpatients with confirmatory laboratory diagnoses of leptospirosis are included; and therefore, problems related to overdiagnosis or gaps in data, common in other available databases [14] were avoided. Moreover, although, we acknowledge that our analysis is depicting a partial view of leptospirosis dynamics, centered on severe cases requiring hospitalization, we would emphasize the expediency of this approach in view of the reliability of such information in depicting leptospirosis epidemiology over a relatively extensive time frame.

Furthermore, we found that the prevalence and incidence rates of hospitalized cases were maintained at a median of 123 cases/year but with elevations every 4 years—for the example of incidence rates, in 2008, 1.45; 2012, 2.45; and 2016, 0.68—with those data corresponding to increases in rainfalls during the same years that elevated temperatures and stagnated water (https://brenp.com/inamhi-anuarios-metereologicos-en-pdf/). During 2020, a publication involving data from the MoH-SIVE-ALERTA system reported an increment in cases of leptospirosis in 2019 (n = 137), but a limited number for 2020 (n = 75) (https://www.salud.gob.ec/wp-content/uploads/2021/02/Leptospira-SE-07.pdf). We expected to also observe the same increase in hospitalizations; nonetheless, only 105 and 47 cases were added to the INEC database during those respective years. We hypothesized that this inconsistency might be a consequence of lockdowns implemented to control the ongoing COVID-19 pandemic. Indeed, multiple health centers were closed, and hospitals were overcrowded treating SARS-CoV-2–infected individuals [22,23], which might have hindered the healthcare system’s ability to treat or attend patients with other pathologies.

Seventy-nine deaths were reported, representing a cumulative case-fatality rate of 3.06%, which is lower than the value reported worldwide, which figure varies from 5% to 15% [1] with a median of 6.95% [2]. This lower case-fatality rate in Ecuador could be associated with the recent improvement in healthcare facilities and coverage of hospitalization throughout the entire country [13]. During outbreaks in the Coast, though, the mortality increased to up to 12% [10]. These low figures also could possibly be related to underreporting, mainly because these data come from hospitalized cases only, neglecting ambulatory cases of leptospirosis from remote populations. Nevertheless, the highest rate of mortality reaches to 0.047 in 100,000 inhabitants in 2001 and 2010.

As reported in other endemic countries, our study demonstrated that more leptospirosis cases occurred in males (*i*. *e*., 65.8%; *p* <0.001), in agreement with previous reports from this country from SIVE-ALERTA data [13,15] indicating the same pattern. Worldwide, adult males are at higher risk of contracting leptospirosis, with those accounting for approximately 80% of the total burden [2], evidence that argues in favour of leptospirosis as an infection with an occupational nature of transmission and sex-related susceptibility. Male and female adults from 25 to 34 years constituted the age group most affected in the present study; the case rates among adults between 20 and 50 years of age are consistently reported as the highest [2]. Usually, adults of this age engage in outdoor activities in the field of agriculture, cattle raising, and fishing; with those activities constituting known risk factors for *Leptospira* spp. infection. The economy of Ecuador depends heavily on agriculture and livestock, with 36% of the population living in rural areas with large extensions of rice fields and banana plantations.

In our study, people living in rural cantons of the tropical regions (*i*. *e*, the Coast and Amazon) were the most affected because abundant water, agriculture and livestock-related activities exponentially increase the transmission and acquisition of *Leptospira* infection. Accordingly, the Coast recorded the highest number of confirmed leptospirosis cases at an average annual incidence rate of 1.12 rising to 2.45 per 100,000 population, a value even higher than the incidence expected by the Pan-American Health Organization [5]. The Manabí province accounted for 39.8% (1029/2584) of the total cases, which also corresponded to half the cases of the Coast followed by Guayas (19.4%; 502/2584), Esmeraldas and Los Rios (7.2%; 186/2584), and Morona Santiago (3.5%; 91/2584). Moreover, Zamora Chinchipe (3%; 79/2584) and Pichincha (6.6%; 171/2584), located in the Amazon and Andes, respectively, also had a high number of cases. In Ecuador, the highest frequency of floods occurred in the rainy season in the Coastal provinces, with Guayas being the most highly affected followed by the Manabí, Los Ríos, Esmeraldas, and El Oro provinces [24]. We should note that rainfall is longer and warmer in the Amazon [16], but the population estimation and density are lower in this geoclimatic region [17]. In a seroepidemiological study on the aetiology of acute undifferentiated febrile illness in the Ecuadorian Amazon, leptospirosis was found in high frequencies [11]. The Andean provinces represented 11.5% (n = 297/2584) of the total cases, with the Pichincha province being the most severely affected. We need to mention that before November 2007, the tropical province of Santo Domingo de los Tsáchilas was considered a canton of the Pichincha province in the Andean region and therefore after 2007, more cases were included in the counts of the Coast. Moreover, certain Andean provinces have cantons traversing the subtropical and tropical biomes; thus, our proposed spatial clusters of hospitalized cases at the canton level would help in the discrimination of a potential environmental risk of infection.

The spatial analysis at the canton level of the present study revealed 12 significant clusters of leptospirosis involving 8 non-overlapping cantons, localized in the central and northern portions of the Coast region and in the southern Amazon basin. The first cluster, found in the Coast, accounted for 31% of inpatients (n = 794/2584). The second cluster registered in the Amazon accounted for 4% of cases (n = 104/2584). The cantons corresponding to both clusters should be key areas for improving the surveillance and control of potentially severe cases of leptospirosis. These ecoregions were also emphasized by Nuñez-González et al. (2020) after using the outpatient SIVE-ALERTA database from 2013–2018 [15].

*Leptospira* spp. infections are known to increase during rainy seasons because of the pathogen’s water-based form of transmission [4]. Our analysis demonstrated a statistical difference in the total number of hospitalizations over three six-month comparisons in the Coast. The environmental conditions within this ecoregion were potentially responsible for the surge of hospitalizations, as has been demonstrated elsewhere [25]. The Ecuadorian Coast is the one most susceptible to environmental changes, as its climatic conditions are impacted by the Pacific Ocean, and meteorologic phenomena such as the El Niño and La Niña Currents can increase flooding and, potentially, the risk of *Leptospira* infection [25,26]. Conversely, a lack of statistical differences among the comparisons in the Andes and Amazon would suggest the possibility that cases might be registered there at any time of the year. Nonetheless, peaks of hospitalizations throughout the Amazon might be driven by local climatic conditions and nonenvironmental determinants of infection such as sanitation and human behavior [18]. Analyses of case numbers in temperate countries might contribute to furthering the evidence for the role of environmental determinants for leptospirosis. In the Andes, the rainy months are less strong than in the tropics [16]. In general, most of the hospitalized cases in the present study were recorded from March to May, coinciding with the rains in both the Coast and Amazon [16]. Higher temperatures and rainfall are optimal conditions for the growth of *Leptospir*a spp. and reservoirs. Barragan et al. (2016) reported that the cases of leptospirosis in Ecuador were strongly associated with rainfall [27]. During the rainy season, flooding is common and leaves ponds and stagnated water in both the tropical rural villages and the towns. Furthermore, inhabitants also use more river water for the purpose of personal hygiene (*e*. *g*., washing and bathing), thus increasing the chance of acquiring *Leptospira* infection. Nevertheless, in a river experiment within a highly endemic area of the Coast (Manabí province), pathogenic *Leptospira* were detected in only 3.7% of the water samples but were present in 22% of the shore soil [28]. Further investigations exploring the role of rivers and water bodies in the transmission of *Leptospira* spp. are accordingly needed to resolve these questions.

This study has certain limitations. First, the analysis of hospitalized cases misses asymptomatic infections or cases with mild symptoms, but instead concentrates on only severe cases. Notwithstanding, within the nosology of the disease, since the milder cases not requiring hospitalization may well be attributable to specific less pathogenic strains of *Leptospira*, the present sampling reflecting the abundance and pathogenicity of the more dangerous forms would have a greater relevance to the basic healthcare considerations underlying public well-being in the country. Moreover, the strength of this dataset comes from the certainty of leptospirosis diagnoses via laboratory confirmations (*i*. *e*., the MAT or double ELISA). Thus, these individuals are no doubt infected with the pathogenic forms of *Leptospira* spp. Second and third, the INEC database neglects important details of leptospirosis diagnosis including the titers of MAT and ELISA tests and the identification of the *Leptospira* spp. and serovars infecting a given patient, which information might be available at local hospitals and did not record the symptomatology of the illness; but that information has definitely not yet been centralized but would be recommended. Finally, potentially some severe cases might be lost due to the lack of sensitive culture and molecular diagnostic methods, which are not available in Ecuadorian public hospitals. Accordingly, the data used here were the best available for conducting this retrospective analytical study.

## Conclusion

In Ecuador, human leptospirosis is a life-threatening disease producing severe symptomatology that needs hospitalization and can be lethal. Leptospirosis is widely endemic and has an apparent seasonal distribution clustered around the central and north of the Ecuadorian Coast and in the southern Amazon basin. Outbreaks of leptospirosis are closely associated with the rainy season, which corresponds to periods following increased rainfall and flooding in the Coastal provinces; for example, in the aftermath of the El-Niño-Southern-Oscillation occurrences. Since Ecuador is located in the Torrid Zone, global warming is likely to continue to alter climatic conditions that might predispose these regions to an increase in leptospirosis risk.

## Supporting information

S1 Table Curated database of hospitalized leptospirosis cases in Ecuador (2000–2020) derived from the National Institute of Statistics and Census (INEC, Spanish) publicly available data (https://www.ecuadorencifras.gob.ec/institucional/home/). (XLSX)
